# Diagnosis of infectious diseases

**DOI:** 10.1099/jmm.0.002055

**Published:** 2025-10-27

**Authors:** Robert J. Shorten, Katharine Hayden, Mathew A. Diggle

**Affiliations:** 1 Lancashire Teaching Hospitals NHS Foundation Trust, Preston, UK; 2 Division of Medical Education, University of Manchester, Manchester, UK; 3 Manchester University NHS Foundation Trust, Manchester, UK; 4 Association for Laboratory Medicine, London, UK; 5 Provincial Laboratory for Public Health (ProvLab), Alberta Precision Laboratories (APL), Alberta Health Services, Edmonton, Canada; 6 University of Alberta Hospital, Edmonton, Canada

Whether supporting an individual patient with an acute infection or responding to an epidemic or even a worldwide pandemic, the role of an effective and efficient method to establish the cause is the cornerstone of the diagnostic pathway. Accurate diagnostic tests are fundamental in the quality of care and to establish the best outcome for patients and the wider population.

As a well-established specialization over the last century, there has been a dramatic development in the advances in a wide range of technologies over the past few decades. These not only directly serve the patients’ needs and diagnose a suspected infection but also facilitate the quality of testing through effective management and oversight.

Despite these continued advances in diagnostic technologies, many patients are still undiagnosed or mis-diagnosed which can lead to ineffective management, treatments and poor outcomes. Consequently, there is a continual need to improve the complete patient and sample pathway, including pre-analytical decision-making, analytical technologies, and post-analytical interpretation and actions. Novel tests that can identify a specific pathogen or, at a minimum, distinguish between different types of bacterial, viral, parasitic or fungal infections, and ideally provide information on ways to effectively treat are becoming increasingly prevalent. This continual improvement leads to improvements in clinical outcomes for patients, effective treatment stewardship, detection and tracking of disease outbreaks, and investigation of unknown pathogens. Moreover, the improvement of regulatory systems and quality practices ensures sustainability in the progression of improvement practices.

The Microbiology Society’s *Journal of Medical Microbiology* has collaborated with the Association for Laboratory Medicine to create this special collection on Diagnosis of Infectious Diseases. In this collection, we provide insightful and impactful articles highlighting a wide range of areas contributing to the continual development of better diagnostic pathways in infection, from novel technical advances and quality systems to the importance of effective infrastructure and leadership.

We have been able to gather some of the leading experts in their areas of specialization to provide their insightful knowledge to this collection. This collection aims to provide a comprehensive platform for the dissemination of cutting-edge research, advancements and innovations across the broad field of diagnosing infectious diseases. Our outcome is to facilitate collaboration among researchers, clinicians and professionals from various disciplines to address critical challenges in accurate and timely diagnosis, surveillance and management of infectious diseases.

At the Microbiology Society and the Association for Laboratory Medicine, we are working together for the benefit of everyone involved in our communities. This collaboration aims to bring together and empower communities of healthcare professionals and scientists for cross-community knowledge exchange and to amplify all our members’ voices, to embed the benefits of laboratory medicine and diagnostics in wider society. We hope this partnership and subsequent collection will continue to facilitate collaboration across disciplines to further research and clinical practice.

This collection aims to showcase the breadth, complexity and importance of diagnostics in infectious diseases and public health, including to a non-specialist audience.

It will also serve as a vital conduit for sharing knowledge, promoting dialogue and accelerating the translation of scientific advancements into tangible improvements in the diagnosis and management of infectious diseases.

